# Supplementary material for: CRISPR/Cas9-mediated excision of ALS/FTD-causing hexanucleotide repeat expansion in C9ORF72 rescues major disease mechanisms in vivo and in vitro
Source: Nat Commun. 2022 Oct 21;13:6286. doi: 10.1038/s41467-022-33332-7 (PMC9587249; doi:10.1038/s41467-022-33332-7)
Supplement: Supplementary file 1 — Supplementary Information [file 41467_2022_33332_MOESM1_ESM.pdf]

Supplementary Figure 1

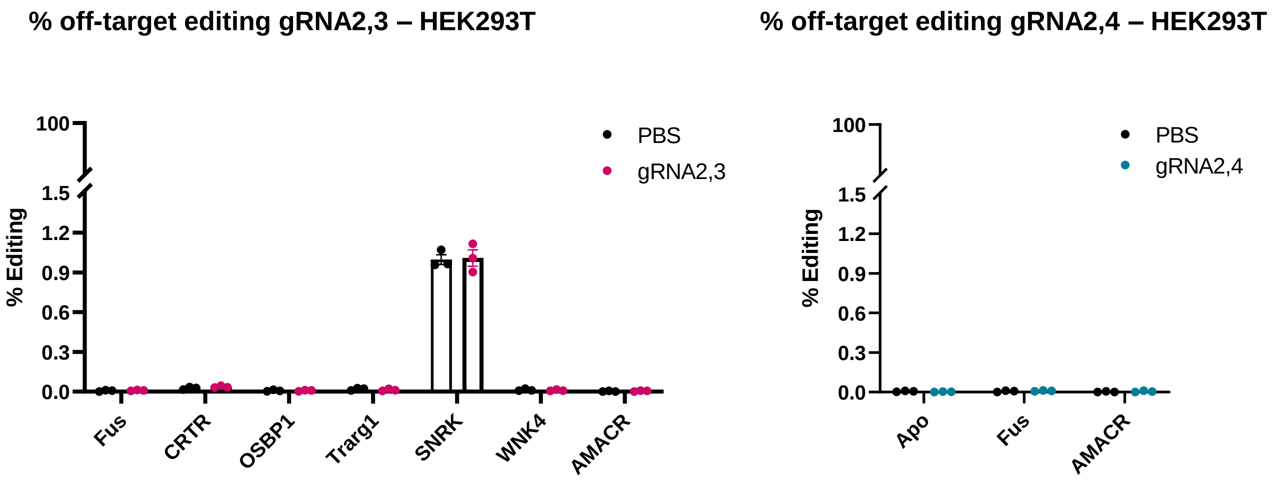

Supplementary Figure 1. CRISPR Amplicon Sequencing results after gRNA2,3 and gRNA2,4 editing in HEK293T cells of coding sequences that had 3 mismatches with gRNA 2, 3 or 4. NGS data was analyzed using CRISPResso2, data represent mean +/- SEM, *n* = 3, no significant differences between treated and untreated cells.

Supplementary Figure 2

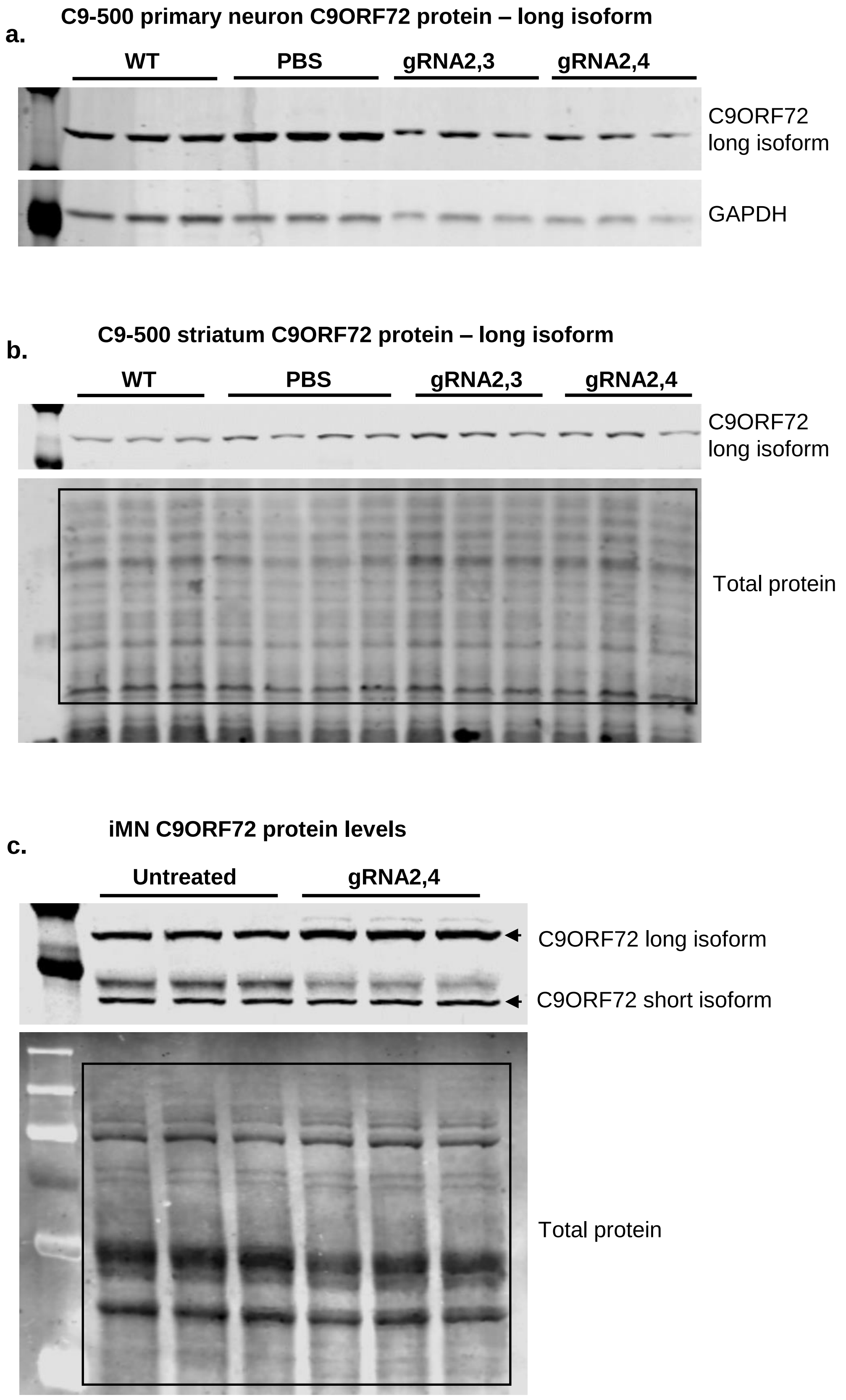

Supplementary Figure 2. Original blots of Western blot. **a.** Original blots of Figure 2g. **b.** Original blots of Figure 3h. **c.** Original blots of Figure 5k.

Supplementary Figure 3

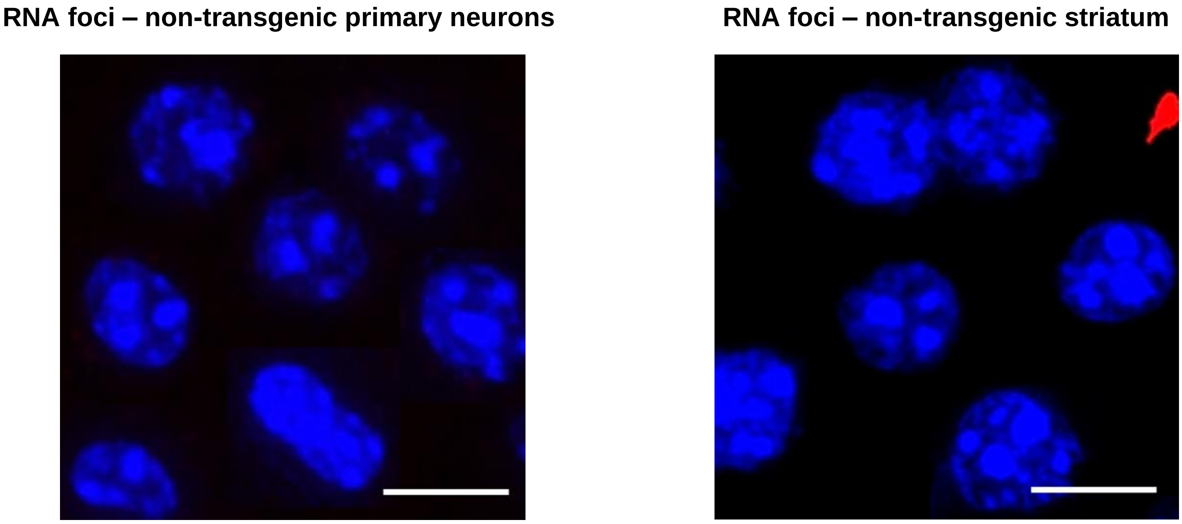

Supplementary Figure 3. Representative images of non-transgenic primary neurons and non-transgenic striatum stained for sense RNA foci using (FISH). Stainings for non-transgenic primary neurons and striatums were performed at the same experiment as Fig. 2d. C9-500 primary neurons and Fig. 3e. C9-500 striatums. to No sense RNA foci were detected in non-transgenic samples. Scale bars represent 10µm.
